# Using supervised machine learning classifiers to estimate likelihood of participating in clinical trials of a de-identified version of ResearchMatch

**DOI:** 10.1017/cts.2020.535

**Published:** 2020-09-04

**Authors:** Janette Vazquez, Samir Abdelrahman, Loretta M. Byrne, Michael Russell, Paul Harris, Julio C. Facelli

**Affiliations:** 1 Department of Biomedical Informatics, University of Utah, Salt Lake City, UT, USA; 2 Computer Science Department, Faculty of Computers and Artificial Intelligence, Cairo University, Giza, Egypt; 3 Vanderbilt University, Nashville, TN, USA; 4 Center for Clinical and Translational Science, University of Utah, Salt Lake City, UT, USA

**Keywords:** Supervised machine learning, deep learning, convolutional neural network, clinical trial recruitment, clinical trial participation

## Abstract

**Introduction:**

Lack of participation in clinical trials (CTs) is a major barrier for the evaluation of new pharmaceuticals and devices. Here we report the results of the analysis of a dataset from ResearchMatch, an online clinical registry, using supervised machine learning approaches and a deep learning approach to discover characteristics of individuals more likely to show an interest in participating in CTs.

**Methods:**

We trained six supervised machine learning classifiers (Logistic Regression (LR), Decision Tree (DT), Gaussian Naïve Bayes (GNB), K-Nearest Neighbor Classifier (KNC), Adaboost Classifier (ABC) and a Random Forest Classifier (RFC)), as well as a deep learning method, Convolutional Neural Network (CNN), using a dataset of 841,377 instances and 20 features, including demographic data, geographic constraints, medical conditions and ResearchMatch visit history. Our outcome variable consisted of responses showing specific participant interest when presented with specific clinical trial opportunity invitations (‘yes’ or ‘no’). Furthermore, we created four subsets from this dataset based on top self-reported medical conditions and gender, which were separately analysed.

**Results:**

The deep learning model outperformed the machine learning classifiers, achieving an area under the curve (AUC) of 0.8105.

**Conclusions:**

The results show sufficient evidence that there are meaningful correlations amongst predictor variables and outcome variable in the datasets analysed using the supervised machine learning classifiers. These approaches show promise in identifying individuals who may be more likely to participate when offered an opportunity for a clinical trial.

## Introduction

Recruitment for clinical trials (CTs) and interventional studies is critical for the evaluation of new pharmaceuticals, therapies and devices. Although CT enrollment has increased by almost 50% from 1996 to 2002, the numbers remain low, especially amongst minorities [1,2]. In a study conducted by the Center for Information and Study on Clinical Research Participation, while 80% of people surveyed expressed a willingness to participate in clinical research, only about 1–2% of Americans participated in CTs annually [3–5]. Methodological concerns could arise from a shortage of participants, and prolonged or inefficient recruitment can have severe economic impacts on a study [4,6]. Therefore, quantitatively understanding and predicting the characteristics of CT participants may provide better approaches to target limited recruitment resources towards individuals most likely to participate [6] or highlight those unlikely to participate in support of representation of the desired population.

Previous studies have analysed the general population’s perception of clinical research and the barriers they face when it comes to volunteering for a CT. For example, in a study by Tramm *et al.* [7], it was found that less than 3% of adult cancer patients participate in CTs. A Harris interactive poll indicated lack of awareness as the main issue for lack of participation [7]. Tramm *et al.* also found a statistically significant relationship between those with knowledge of CTs and those willing to participate, with 60% of those with knowledge about trials going on to enroll [7]. Similar studies have delineated other issues that inhibit people from participating in CTs. These issues include lack of interest, lack of transportation or household location that is too far from the trial location, lack of time, fear of emotional distress, fear of how it may affect their health, media-related factors, privacy concerns and a general lack of trust towards medical research [1–3,5,6,8–11]. There is also evidence showing that demographic characteristics are influencing factors in enrollment, with higher rates of refusal found in participants with low income, low education and low health awareness [6]. Studies have found that a participant’s perceptions of CTs, age, gender, race, ethnicity and socioeconomic status all impact their rate of enrollment in CTs [1,2,6,8–11].

Community engagement, educational meetings, onsite recruitment and referrals from clinicians are possible approaches to lower these barriers, though these efforts require additional resources from researchers [6,8,10]. The effect of message framing, evaluated by Balls-Berry *et al.* [12], found that there was no advantage in using either gain- or loss-framed messages. Although attitudes towards participation in research studies were favourable, only one out of four participants who answered the survey on attitudes towards research participation registered for further clinical research, indicating deeper issues [12]. Flood-Grady *et al.* [13] studied the effectiveness of various communication strategies for enrolling patients in CTs. They utilized registries to find participants and found that combining both passive and active methods of communication resulted in higher enrollment numbers [13]. Active methods, such as telephone calls or personal visits, have been found to produce better results than passive methods, such as sending letters or emails, but this comes at a higher cost. Being able to find participants who would be more responsive to participating in CTs and actively recruiting these participants would help lower the costs of recruitment in CTs by targeting existing limited resources towards candidates who are more likely to participate. Alternatively, finding patients who are unlikely to participate and targeting recruitment resources to these patients may produce cohorts that are more representative.

Most studies discussed above have used qualitative or semi-quantitative research methods. The goal of this project was to determine if modern analytical techniques could be used to identify characteristics of individuals likely to participate in CTs. Specifically, we analysed a de-identified dataset from ResearchMatch participants. The dataset included demographic variables (age, gender, race, ethnicity, tobacco use), medical conditions, any current medications, geographic preference (self-reported distance willing to travel from home) and de-identified logs of ResearchMatch utilization to postulate underlying associations about factors influencing likelihood to participate in a clinical trial [5]. Although previous studies have looked at similar factors, such as race, gender, ethnicity, socioeconomic status and education and analysed those in association with CT participation, to our knowledge none of these studies have used supervised machine learning classifiers to assess the likelihood of participating in a CT [14–17]. We also used a deep learning approach to analyse our dataset and demonstrate that deep learning techniques are not only feasible but may also provide better results than supervised machine learning classifiers in identifying enrollees who are more likely to participate in a clinical trial.

### ResearchMatch, an Online Clinical Trial Registry

In 2008, the Clinical and Translational Science Awards Consortium decided to address the challenges facing recruitment and set five strategic goals to ‘improve all processes related to the development, approval, activation, enrollment, and completion of clinical trials’ [5]. ResearchMatch, built by Vanderbilt University Medical Center in late 2009, was proposed as a national registry for CTs available to both researchers and participants across the nation to make it easier to bring them together, rather than relying on recruiting agents to find participants.

ResearchMatch works by allowing individuals from the general public to self-register and express an interest in participating in clinical studies and trials. Registration includes a short survey process where individuals self-report medications, medical conditions and demographic information about themselves. Once registered, individuals are deemed potential volunteers in the ResearchMatch system and available to be ‘found’ by a potential researcher whenever they fit basic inclusion and exclusion criteria for a specific study. Once they are deemed a potential candidate for a specific study, ResearchMatch research teams initiate contact by sharing introductory information about the study in a privacy preserving workflow designed gauge interest from the volunteer. Once contacted, the potential volunteer has the choice to ignore or signify further interest and if interested, a ‘match’ is made between researcher and volunteer to discuss additional details of the study and, whenever appropriate, consent into the study. Researchers can be from any ResearchMatch participating medical centre or institution and the cohort identification, messaging and eventual specific contact to interested individuals is completely self-service. The ResearchMatch system collects activity logs of the aforementioned contact transactions, and these logs were completely de-identified for use in the current study.

In 2012, ResearchMatch performed an initial assessment of registry activity and utility. Approximately 20% of those contacted to gauge interest in a clinical research opportunity responded with interest. Out of the volunteers that were not interested, 7.3% gave feedback as to why they had decided against participation. The reasons given included: ‘not thinking they met criteria’, ‘not interested’, ‘lack of time’, ‘distance’ and ‘commitments to other studies’.

### Previous Work using Machine Learning for Clinical Trial Studies

Machine learning techniques are often used to gain novel insights and a deeper understanding of data by discovering patterns and trends that are not apparent. The successful implementation of machine learning can provide novel biomedical and health care knowledge beyond the scope of what statistical methods could find, as several studies and examples have shown [18,19]. Previous work by Xiong *et al.* applied a deep learning approach to find patients in an electronic health record that may be a good fit for CTs. They used a hierarchical neural network and applied it to identify patients who satisfied specific criteria for clinical trial studies [20]. In our study, we decided to implement supervised machine learning classifiers and a deep learning model to data from an online registry populated by individuals showing *a priori* interest in participation in clinical studies. Other studies have used Logistic Regression (LR) to analyse characteristics of clinical trial participants (and lack of) [1]. However, more robust machine learning classifiers, such as Random Forest Classifier (RFC), and deep learning methods have rarely been used for the prediction of those more likely to participate in a clinical trial, especially with more features incorporated into a predictive model.

## Methods

**Fig. 1.**
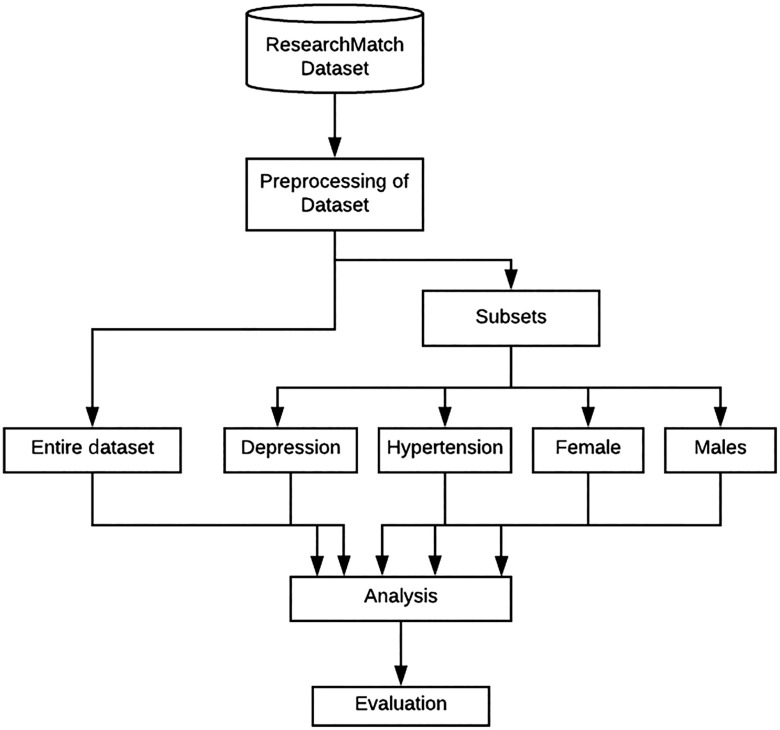
Pipeline of method for analysis.

We trained and tested supervised machine learning classifiers and a deep learning model on a large de-identified dataset obtained from Vanderbilt University Medical Center consisting of data from volunteer registrants on ResearchMatch. The study methods consisted of the following steps: (1) preparation of the ResearchMatch dataset, (2) training and tuning our machine learning classifiers and deep learning model and (3) evaluating the classifiers and deep learning model using precision, recall and area under the curve (AUC) (Fig. 1). The study was deemed exempt by both the IRBs of the University of Utah and Vanderbilt University Medical Center.

### Preparation of the ResearchMatch Dataset

The dataset used in this study consisted of two different de-identified information sources gathered from ResearchMatch. The dataset was placed in a protected environment within the University of Utah Center for High Performance Computing, where the data analysis took place [21].

The initial information source consisted of contact IDs, volunteer IDs, contact date, a unique study ID and a response from the potential volunteer consisting of either a ‘yes’ or a ‘no’ determining whether they were interested in a study. If an individual was missing a response to their interest in the study, we placed ‘no’ as the response since a lack of response likely indicated no interest in the study or no interest in participating in ResearchMatch. We also ensured that there was no significant covariate imbalance between the two by performing a standardised difference analysis between the missing values and the variables of the individuals that replied with ‘no’ (Supplementary Table S1). The ‘response’ column is what we used as our outcome label. The second information source consisted of selected self-reported volunteer characteristics, including a system-generated participant linkage ID, age, race, ethnicity, whether they were a veteran, gender, tobacco use, whether a user was a twin, the state a user originated from, whether they were operating as themselves or as a guardian for someone else in their household, the distance a user was willing to travel for a study, medical conditions, medications, the last login to ResearchMatch and, lastly, how the user indicated learned about ResearchMatch during their initial joining of the registry. All participant and researcher identifiers were omitted, and all dates were shifted for de-identification before receipt of information sources.

The two information sources were joined into a single dataset using R 3.2.4 [22]. We used the matching volunteer IDs between the two sources to merge, which resulted in a final dataset for analysis with 20 features and 841,377 rows. Each row in the dataset represented a specific researcher-initiated inquiry about a specific study opportunity to an individual in the ResearchMatch population of potential volunteers. On average, each individual received eight invitations (mean of 8.166 and standard deviation of ±8.311) to a unique study, indicating a repeat of certain individuals within our dataset with differences in their response, the study they were contacted for and the date they were contacted on. The median for invitations per individual was 6, with a 25th percentile of 3 and a 75th percentile of 10. The minimum amount of invitations an individual received was 1, while the maximum was 261. Although subjects received multiple invitations, we did not account for the dependency that could arise from this. Missing answers were treated as their own category and coded as such within the dataset.

### Data Analysis

Associations between predictor variables and the outcome variable, ‘response’, were analysed using R 3.2.4 to complete all of the statistical analyses [22]. We used the chi-square test to measure association for categorical variables and Wilcoxon rank tests (t-test) for the association between continuous variables [23].*P* values < 0.05 were considered statistically significant. We also measured the standardized differences between variables for both ‘yes’ and ‘no’ outcomes (see Results section) [24]. We estimated multicollinearity by calculating variance inflation factors (vif) [25]. Any vif over 5.0 was removed for our final dataset [23]. A vif detects multicollinearity, which is when a correlation exists between predictors in a model. Usually a score higher than 5.0 indicates that there is high correlation amongst predictor variables, and the model may be less reliable. Variables with a high vif are usually removed in order to ensure machine learning models works as needed [23].

After the initial analysis, it was also decided to create new derivates of the dataset using the top two conditions reported by individuals, hypertension and depression, and by gender, males and female, to evaluate if users of ResearchMatch with certain common conditions or of different genders were more likely to show interest in participating in a study.

The four datasets were preprocessed to test for the *P* value between our outcome variable, the responses, and the predictor variables, as well as multicollinearity, before beginning our analysis using supervised machine learning classifiers and the deep learning method [23].

### Training and Testing Datasets

For our machine learning classifiers and deep learning model, we created and used five total datasets. We used a dataset with the entire 841,377 instances and with the following information, which made up each feature or column: contact date, study type, contact type, population type, when the study was created (month/year), institution the study was from, age at account created, race of user, ethnicity, veteran status, gender, tobacco use, whether a user was a twin, state the user originated from, whether they were operating as themselves or as a guardian for someone else in their household, has a condition, is taking a medication, if a guardian account was created, the last login to ResearchMatch, how they learned about ResearchMatch and responses.

The depression dataset consisted of 103,664 instances, and the hypertension dataset consisted of 81,525 instances. These subsets of the dataset were created by counting the different Unified Medical Language System (UMLS) concepts within our dataset and choosing the top two concepts with the most counts. We then created the subsets by choosing all rows with the relevant UMLS concept mentioned under ‘medical condition’. Both of these datasets consisted of 17 features, with only the features ‘has conditions’ and the conditions list removed since those were used to create the subsets. For gender, the female dataset consisted of 627,480 instances, and the male dataset consisted of 210,138 instances. These subsets were created by simply separating the dataset by male and female; both of these had 18 features per participant.

These datasets were then hot encoded, or binarised by features, to be more easily readable by our supervised machine learning classifiers. Hot encoding allows for categorical variables to be better understood by converting them to binary features. The datasets were also split 80% for training and 20% for testing.

### Machine Learning Classifiers and Deep Learning Model

The supervised machine learning classifiers used in this analysis were the following: LR, Decision Trees (DT), RFC, ABC, Gaussian Naïve Bayes and K-Nearest Classifier. These classifiers were implemented using Python 3.5.2, as well as the Scikit-learn libraries, and Chocolate, a python library for hyperparameter optimization [26–28]. Precision, recall, accuracy and AUC scores of the classifiers were used to evaluate their efficacy [23].

We incorporated a deep learning approach in our study to try extracting information from hidden relationships that may exist within the data, as well as the large and heterogenous dataset to be analysed. Supervised machine learning tasks require datasets that have representations, or features; however, it is often difficult to know which features should be extracted [29]. They also require domain expertise and human intervention. Deep learning helps solve this issue of representation by building complex concepts out of simpler concepts through the process of multiple layers of similar functions [30,31]. Deep learning is also more flexible, eliminates need for domain expertise and usually obtains higher accuracy than traditional supervised machine learning classification. Lastly, deep learning also tends to be more scalable versus traditional machine learning that may lose performance or converge as datasets get larger. This would make it easier to implement our model into production in the future and allow it to be used on larger datasets without much of an issue.

For the deep learning implementation of our analysis, we used TensorFlow 1.12 as the backend, with Keras 2.2.4 to create our deep learning models [32,33]. Talos, a python library for hyperparameter optimization in Keras, was used to tune the deep learning model [34]. We used a 1-dimensional Convolutional Neural Network (1DCNN) for this analysis and accuracy, AUC, precision and recall scores were used to measure the performance of the model. After running a python script with the Chocolate library for hyperparameter optimisation, results were obtained for the best-performing hyperparameters for each predictive model.

As previously stated, the supervised machine learning classifiers used for this analysis consisted of LR, DT, Gaussian Naïve Bayes (GNB), K-Nearest Neighbor Classifier (KNC), ABC and a RFC from the Scikit library [27]. The optimal hyperparameter for the DT classifier was a max depth of 72. The RFC performed best at a max depth of 320, a minimum leaf sample of 10, a minimum sample split of 32 and 394 trees. The KNC had a leaf size of 75, 2 neighbours, used Euclidian distance as the power parameter (*P* = 2) and used distance for the weights. The ABC performed best at a learning rate of 1 and a maximum number of 390 estimators. The supervised machine learning classifiers were run using 10 cross-fold validation. The scores were averaged across the folds before using the validation dataset on the classifier.

We also used a 1DCNN for our deep learning part of the analysis. After running our program with various hyperparameters using Talos [34], the optimal hyperparameters for the dataset were found. The network consisted of four layers. The first three contained a ReLU activation, with the last layer containing a Sigmoid function as the activation function. Binary Cross Entropy was used to measure for loss. We used the Adamax function as the optimizer for our network. The first two layers of our network contained 64 neurons, followed by a layer with 128, and a final layer with 1 for the output [33]. We ran our model for 1000 epochs.

## Results

A descriptive analysis of the final dataset used for the analysis is available in Table 1.

**Table 1.** Descriptive statistics of ResearchMatch dataset

Demographic/Health Data	Total
Total instances	841,377
Unique users	102,510
Age, mean (SD)	35.67 (E±16.45)
Gender	
Male	28,579 (27.88%)
Female	73,627 (71.82%)
Transgender	293 (0.29%)
No Answer	11 (0.01%)
Response *(Response = every different study email sent to an individual)*	
Yes (%)	420,688 (50%)
No (%)	154,018 (18.31%)
No Answer (%)	266,670 (31.69%)
Race	
American Indian/Alaska Native (%)	692 (0.68%)
Asian (%)	3693 (3.60%)
Black or African American (%)	11,628(11.34%)
Multiracial (%)	4847 (4.73%)
Native Hawaiian/Pacific Islander (%)	215 (0.21%)
Other (%)	2929 (2.86%)
White (%)	78,492 (76.57%)
No Answer (%)	14 (0.01%)
Ethnicity	
Hispanic (%)	7795 (7.60%)
Non-Hispanic (%)	94,626 (92.31%)
No Answer (%)	89 (0.09%)
Tobacco Use	
Yes (%)	16,540 (16.14%)
No (%)	85,933 (83.83%)
No Answer (%)	37 (0.03%)
VetStatus	
Non-Veteran (%)	77,545(75.65%)
Veteran (%)	4433 (4.32%)
No Answer (%)	20,532 (20.03%)
Multiple Birth Status	
Single (%)	100,208 (97.75%)
Twin (%)	2172 (2.12%)
Triplet (%)	98 (0.10%)
No Answer (%)	32 (0.03%)
Medical conditions	
No medical conditions (%)	35,495 (34.63%)
Reported medical conditions (%)	66,559 (64.93%)
No Answer (%)	456 (0.44%)
Most frequent conditions	
1 C0344315 Depression	94,626 (92.31%)
2 C0020538 Hypertension	76,631 (74.75%)
3 C1963064 Anxiety	51,658 (50.39%)
Medication usage	
No medication use (%)	38,329 (37.39%)
Reported medication use (%)	63,296 (61.75%)
No Answer (%)	885 (0.86%)
Most frequent medications	
1 C0978787 Multivitamins tab	29,293 (28.58%)
2 C0162723 Zyrtec	16,163 (15.77%)
3 C0728762 Synthroid	16,223 (15.83%)
Top 3 States	
1 OH (%)	12,751 (12.44%)
2 TN (%)	7953 (7.76%)
3 NY (%)	7803 (7.61%)
Willing to travel (in miles)	
0 (%)	296 (0.29%)
50 (%)	43,219 (42.16%)
100 (%)	17,902 (17.46%)
200 (%)	22,445 (21.90%)
300 (%)	2006 (1.96%)
1000 (%)	16,642 (16.23%)
Charge	
Guardian (%)	6413 (6.26%)
Self (%)	96,097 (93.74%)
How learn	
Facebook-Advertisement (%)	738 (0.72%)
From a friend/colleague (%)	6960 (6.79%)
From an organization (%)	16,748 (16.34%)
From my physician (%)	1198 (1.17%)
Health fair (%)	554 (0.54%)
News release (%)	1933 (1.89%)
Other promotion (%)	10,039 (9.79%)
RM code (%)	20,134 (19.64%)
Search Engine – Google (%)	14,957 (14.59%)
No Answer (%)	29,249 (28.53%)

A chi-square test was used to measure the association for categorical variables and a Wilcoxon rank test (*t*-test) for the association between continuous variables with *p* values < 0.05 considered as statistically significant. All variables had a *p* value < 0.05. We also looked at the standardised differences for each variable between those that responded ‘yes’ and those that responded with ‘no’. All of the standardised differences were < 0.01. We estimated multicollinearity by calculating variance inflation factors (vif) and any vif over 5.0 was removed for our final dataset (Table 2). No variables were removed due to multicollinearity.

**Table 2.** Standardized differences (SMD) and multicollinearity values for ResearchMatch dataset. Standardized differences are comparisons between ‘yes’ and ‘no’ responders

Variable	SMD	Multicollinearity
Contact_date	0.028	1.076947
Age_at_account_created	0.001	1.148036
Race	0.005	1.098512
Ethnicity	0.004	1.096648
Vetstatus	0.012	1.516087
Gender	0.003	1.092575
Tobacco	0.008	1.048358
Twin	0.001	1.060303
State	0.012	1.025191
Parentstatus	0.001	1.389189
Willing_to_travel	0.002	1.348529
Charge	0.004	1.089152
Has_conditions	0.007	2.467756
Has_meds	0.009	2.029087
Guardian_account_created	0.032	1.983791
Last_login	0.026	1.735373
How_learn	0.017	1.148880
Condition	0.001	2.071828
Medication	0.001	1.716581

Table 3 shows the results for the final dataset created from the original ResearchMatch datasets. The best-performing algorithm on the ResearchMatch dataset in predicting the likelihood of an individual expressing interest in participating in a clinical trial was the deep learning method, the CNN, with an AUC of 0.8105 and an accuracy of 75%. The accuracy indicates that 75 out of 100 times, the CNN algorithm is likely to predict correctly whether an individual would show interest in a study or not. The CNN also achieved a recall, or true positive rate, of 0.7738, which shows the number of people the algorithm detected as showing interest in a clinical trial study out of all individuals in our dataset that responded with a positive answer, or show of interest in a study. Similarly, the precision, or positive predictive value, was 0.7371, which indicates how many of the positive predictions made by the algorithm were actually positive when compared with the dataset.

**Table 3.** Results for ResearchMatch dataset

Machine Learning Classifiers	AUC – Validation	AUC – Testing	Accuracy	Recall	Precision
CNN	0.8748	0.8105	0.7483	0.7738	0.7371
RFC	0.7284	0.7288	0.7284	0.7306	0.7271
KNC	0.7094	0.7091	0.7095	0.6037	0.7653
Decision Tree	0.7027	0.7047	0.7027	0.7179	0.6963
ABC	0.6804	0.6803	0.6804	0.6817	0.6796
LR	0.6394	0.6383	0.6394	0.6511	0.6358
GNB	0.5895	0.5872	0.5895	0.6743	0.5762

RFC, Random Forest Classifier; ABC, Adaboost Classifier; KNC, K-Nearest Neighbor; GNB, Gaussian Naïve Bayes; LR, Logistic Regression; CNN: Convolutional Neural Network

This was followed in performance by the RFC, which had an AUC of 0.7288 and a 73% accuracy. The next highest was the KNC with a 0.7091 AUC and a 71% accuracy. The deep learning model outperformed the supervised machine learning classifiers in nearly every category, except for precision, where the CNN was outperformed in precision by our KNC (0.7371 vs 0.7653).

Subsets of the dataset were created to see if performance of our supervised machine learning classifiers and deep learning model would increase, but there was not much of a difference between our conditions subsets versus the entire dataset analysed when compared. The CNN performed approximately the same for both the depression and the hypertension dataset, with an AUC of 0.7970 for the depression dataset and an accuracy of 73% and an AUC of 0.7848 with an accuracy of 72% for the hypertension dataset. The RFC continued to perform strong for both the depression and hypertension dataset, but rather than the K-Nearest Neighbor, our DT performed the third best with an AUC of 0.7171 and accuracy of 72% for the depression dataset and an AUC of 0.7267 and accuracy of 73% for the hypertension dataset (Supplementary Tables S2 through S3).

For the female and male subset datasets, the CNN once more outperformed the other supervised machine learning classifiers, and the performance for the subsets was slightly better than for the entire set. For the female dataset, an AUC of 0.8012 and an accuracy of 74% were observed. For the male dataset, an AUC of 0.8216 and an accuracy of 76% were observed. The RFC was the next best performing supervised machine learning classifier, with an AUC of 0.7210 and accuracy of 72% for the female dataset and an AUC of 0.7313 and accuracy of 73% for the male dataset. The DT was the third best performing machine learning classifier for both datasets, with an AUC of 0.7019 and accuracy of 70% for the female dataset and an AUC of 0.7252 and an accuracy of 73% for the male dataset (Supplementary Tables S4 through S5).

## Discussion

To our knowledge, this study is the first to report on using robust supervised machine learning classifiers as well as a deep learning model to analyse the interest in participation for individuals on an online CT registry. The results show sufficient evidence that there are meaningful correlations amongst predictor variables and outcome variable (expression of interest) in the datasets analysed using the supervised machine learning classifiers. This is true for almost all classifiers tested here, but it is especially evident with the analysis using a deep learning model. The results achieve a significant accuracy of over 80% in predicting the outcome, indicating potential for future use of this type of analysis in assessing which individuals may be most interested to participate in a clinical trial.

The deep learning model may have performed better due to the ability of deep learning models to pick up on patterns that may have previously been ignored by supervised machine learning classifiers, as well as their robustness for noisy datasets. When performing an error analysis with the validation datasets, the deep learning model produced the correct output in 0.8% of the rows. In the testing set, out of 117,003 predictions, the CNN was the only correct prediction on 953 of the rows, with 658 belonging to the ‘yes’ response and 295 belonging to the ‘no’ response. Using AutoML also allowed us to look at the weights of the CNN. The features given the most weight within the CNN from the dataset included age, state, how someone learned about ResearchMatch, an individual’s willingness to travel, as well as race and parent status. It is likely that those features were given a higher weight due to how often certain results appeared in the datasets (e.g., 76% of race consisted of white, 42% of people were willing to travel 50 miles, etc.).

As we also saw in the results, creating subsets of our dataset did not produce better results. The depression and hypertension dataset, as well as the female and male datasets, did not deviate much in results from our main dataset. It could be possible that there may not be much distinction amongst participants with conditions, as well as participants of different genders.

Several limitations exist within our study. First, there is a lack of prior research using supervised machine learning classifiers and deep learning methods for this type of analysis, which means there are no other studies to compare our results. Future work is needed to better assess the accuracy of our supervised machine learning classifiers and deep learning model. Second, we interpreted a lack of response to interest in a study as a ‘no’. While this may be the case, a lack of response could also be due to a change in emails or other events not having to do with showing interest in a study or ResearchMatch. Our data source also likely had selection bias since those who self-registered onto ResearchMatch were already predisposed to an interest in participating in research studies. We also did not account for dependencies that could arise from multiple invitations being sent to one person. The max number of invitations a person did receive was 261, less than 0.03% of our dataset which we did not believe would skew our results. Another limitation was the lack of interpretability for this study, especially in the deep learning model, since all variables were looked at for prediction and appear to contribute in discernible manner, a common feature of using ANN for prediction models [30]. Future studies looking at the weights of certain variables in the deep learning model could potentially lead to better interpretability of which variables were most important for predicting the outcome in this study. Performing an error analysis of our model, looking for bias and changing the layers of our model accordingly would also produce more robust results. Although we used a convolutional deep learning network and optimising strategies for our analysis, there are other possible deep learning methods that we could test out on our dataset as well which could lead to improved results.

Lastly, it should be considered that although using these methods may increase the efficient use of resources dedicated to CT recruitment, unexpected biases may be introduced into the CT cohort. Further analysis of feature importance in the algorithms is necessary in order to ensure that bias is not occurring in any one category of our variables (e.g. increasing the chance of likelihood to show interest in a study if an individual has created a guardian account). This issue should be carefully considered before actual implementation of the methods discussed here.

## Conclusion

We used supervised machine learning classifiers, as well as a deep learning model, to see if we could determine characteristics of de-identified individuals from an online clinical trial registry more likely to express interest in a clinical trial. While this does not necessarily indicate participation, it is a good first step for researchers with limited resources to attain a cohort of qualifying participants more likely to participate in a clinical trial. Overall, our classifiers performed relatively well with our deep learning model performing better than other approaches at determining which individuals were most likely to either show interest or a lack of interest in a research study. However, future work is still needed to investigate further strategies that could be applied to our analysis and produce more robust results. In this study, deep learning was shown to be a promising approach in identifying individuals more likely to participate in a clinical trial and could further be used for recruitment resources to target those individuals more actively.

## Supplementary material

For supplementary material accompanying this paper visit https://doi.org/10.1017/cts.2020.535. click here to view supplementary material
