# Supplementary material for: Using supervised machine learning classifiers to estimate likelihood of participating in clinical trials of a de-identified version of ResearchMatch
Source: J Clin Transl Sci. 2020 Sep 4;5(1):e42. doi: 10.1017/cts.2020.535 (PMC8057403; doi:10.1017/cts.2020.535)
Supplement: Supplementary file 1 [file S205986612000535Xsup001.docx]

**Supplementary Materials**

**Supplementary Table S1.** Standardized differences (SMD) for comparisons between individuals with missing values and individuals that responded with ‘no’.

| **Variable** | **SMD** |
| --- | --- |
| Contact_date | 0.012 |
| Age_at_account_created | 0.004 |
| Race | 0.004 |
| Ethnicity | 0.002 |
| Vetstatus | 0.001 |
| Gender | 0.002 |
| Tobacco | 0.001 |
| Twin | 0.002 |
| State | 0.011 |
| Parentstatus | 0.002 |
| Willing_to_travel | 0.003 |
| Charge | 0.002 |
| Has_conditions | 0.003 |
| Has_meds | 0.004 |
| Guardian_account_created | 0.014 |
| Last_login | 0.015 |
| How_learn | 0.004 |
| Condition | 0.003 |
| Medication | 0.002 |

**Supplementary Table S2.** Results for ResearchMatch depression dataset. RFC: Random Forest Classifier; ABC: Adaboost Classifier; KNC: K-Nearest Neighbor; GNB: Gaussian Naïve Bayes; LR: Logistic Regression; CNN: Convolutional Neural Network.

| ML Classifier | AUC - Validation | AUC - Testing | Accuracy | Recall | Precision |
| --- | --- | --- | --- | --- | --- |
| CNN | 0.7939 | 0.7970 | 0.7280 | 0.7397 | 0.6642 |
| RFC | 0.7260 | 0.7288 | 0.7308 | 0.6845 | 0.7341 |
| Decision Tree | 0.7248 | 0.7171 | 0.7247 | 0.7262 | 0.7061 |
| KNC | 0.7046 | 0.7034 | 0.7101 | 0.5809 | 0.7560 |
| ABC | 0.6726 | 0.6777 | 0.6790 | 0.6470 | 0.6700 |
| GNB | 0.6013 | 0.6021 | 0.6079 | 0.4531 | 0.6234 |
| LR | 0.5995 | 0.5990 | 0.6022 | 0.5227 | 0.5955 |

**Supplementary Table S3.** Results for ResearchMatch hypertension dataset. RFC: Random Forest Classifier; ABC: Adaboost Classifier; KNC: K-Nearest Neighbor; GNB: Gaussian Naïve Bayes; LR: Logistic Regression; CNN: Convolutional Neural Network.

| ML Classifier | AUC – Validation | AUC - Testing | Accuracy | Recall | Precision |
| --- | --- | --- | --- | --- | --- |
| CNN | 0.7842 | 0.7848 | 0.7242 | 0.7577 | 0.6992 |
| RFC | 0.7253 | 0.7311 | 0.7311 | 0.7320 | 0.7311 |
| Decision Tree | 0.7208 | 0.7267 | 0.7267 | 0.7394 | 0.7215 |
| KNC | 0.7165 | 0.7067 | 0.7066 | 0.5970 | 0.7651 |
| LR | 0.6756 | 0.6687 | 0.6686 | 0.6615 | 0.6716 |
| ABC | 0.6747 | 0.6720 | 0.6720 | 0.6710 | 0.6728 |
| GNB | 0.6022 | 0.6002 | 0.6001 | 0.4452 | 0.6457 |

**Supplementary Table S4.** Results for ResearchMatch female dataset. RFC: Random Forest Classifier; ABC: Adaboost Classifier; KNC: K-Nearest Neighbor; GNB: Gaussian Naïve Bayes; LR: Logistic Regression; CNN: Convolutional Neural Network.

| ML Classifier | AUC – Validation | AUC - Testing | Accuracy | Recall | Precision |
| --- | --- | --- | --- | --- | --- |
| CNN | 0.8029 | 0.8012 | 0.7367 | 0.7602 | 0.6711 |
| RFC | 0.7216 | 0.7210 | 0.7216 | 0.7210 | 0.7211 |
| KNC | 0.7037 | 0.6988 | 0.7039 | 0.5937 | 0.7604 |
| Decision Tree | 0.7022 | 0.7019 | 0.7005 | 0.7142 | 0.6966 |
| ABC | 0.6757 | 0.6784 | 0.6757 | 0.6764 | 0.6746 |
| LR | 0.6316 | 0.6306 | 0.6316 | 0.6389 | 0.6288 |
| GNB | 0.5889 | 0.5891 | 0.5889 | 0.6074 | 0.5848 |

**Supplementary Table S5.** Results for ResearchMatch male dataset. RFC: Random Forest Classifier; ABC: Adaboost Classifier; KNC: K-Nearest Neighbor; GNB: Gaussian Naïve Bayes; LR: Logistic Regression; CNN: Convolutional Neural Network.

| ML Classifier | AUC – Validation | AUC - Testing | Accuracy | Recall | Precision |
| --- | --- | --- | --- | --- | --- |
| CNN | 0.8187 | 0.8216 | 0.7575 | 0.7726 | 0.6966 |
| RFC | 0.7262 | 0.7313 | 0.7313 | 0.7291 | 0.7327 |
| Decision Tree | 0.7196 | 0.7252 | 0.7252 | 0.7403 | 0.7190 |
| KNC | 0.7165 | 0.7067 | 0.7066 | 0.5969 | 0.7652 |
| LR | 0.6791 | 0.6713 | 0.6713 | 0.6695 | 0.6723 |
| ABC | 0.6747 | 0.6720 | 0.6720 | 0.6710 | 0.6728 |
| GNB | 0.6017 | 0.6009 | 0.6007 | 0.4428 | 0.6479 |
